# Supplementary material for: Are carbon emissions trading and green financial instruments synergistic? -Comprehensive quantitative research based on content analysis
Source: PLoS One. 2024 Mar 7;19(3):e0298601. doi: 10.1371/journal.pone.0298601 (PMC10919601; doi:10.1371/journal.pone.0298601)
Supplement: S1 Data — (ZIP) [file pone.0298601.s001.zip › Data/地级市Z_F工作报告.zip!/║■─╧/╥μ╤⌠╩╨/└┤╫╘╣1⁄2╓┌║┼í╛┬φ┐╦╩2╛▌═°í┐_2016.docx]

各位代表：

现在，我代表市人民政府向大会作工作报告。请各位代表审议，并请各位政协委员和其他列席人士提出意见。

“十二五”主要成就和2015年工作回顾

一、“十二五”主要成就

“十二五”是我市发展进程中极不平凡的五年。面对国内外复杂形势和一系列重大挑战，全市上下在市委的坚强领导下，积极应对，抢抓机遇，开拓进取，经济社会发展取得了新的成绩。

这五年，是我市经济总量不断扩大、综合实力明显跃升的五年。2015年，全市实现生产总值1353亿元（预计数，下同），是2010年的1.7倍，年均增长11%；完成一般公共预算收入104.6亿元，是2010年的2.3倍，年均增长18.4%；完成固定资产投资1221亿元，是2010年的3.6倍，年均增长29.1%；实现社会消费品零售总额574亿元，是2010年的2倍，年均增长15%；实现规模工业增加值554亿元，是2010年的1.9倍，年均增长13.6%。

这五年，是我市产业结构不断优化、质量效益明显提高的五年。三次产业结构由2010年的22.8:40.5:36.7调整为2015年的18.3:42.4:39.3。财政总收入占生产总值比重为7.7%，比2010年提高1.3个百分点。规模工业企业实现利税总额140亿元，是2010年的2.3倍。创新驱动能力不断增强，高新技术企业数量和增加值均较2010年实现翻番。4家企业成功上市，8家企业在新三板挂牌。单位规模工业增加值能耗累计下降49.5%。

这五年，是我市基础设施不断夯实、城乡面貌明显变化的五年。累计完成交通建设投资433.5亿元，是“十一五”的4.3倍。高速公路实现通车里程108.6公里，在建里程327.6公里，益阳成为全省高速公路建设主战场。石长铁路复线建成通车，扩建和新建干线公路408公里，建成农村公路3032公里。完成水利建设投资112亿元。解决了240.1万农村人口的饮水安全问题。光网覆盖60%的行政村，互联网宽带普及率达32%。完成城镇基础设施投资338亿元，城镇化率由39.9%提高到46.2%，成功创建国家卫生城市、国家森林城市、国家优秀旅游城市、全国双拥模范城市；农民集中居住区、新农村示范村和美丽乡村建设取得成绩。

这五年，是我市改革开放不断深化、发展活力明显增强的五年。党的十八大以来，我市共完成9大类117项改革事项，承担了12项省级以上改革试点任务。特别是农村综合配套改革、行政审批制度改革等走在全省前列。累计招商引资形成固定资产投资1407亿元，年均增长15.5%，实际利用外资年均增长13%，成功引进了中核集团等一批战略投资者。引进银行业金融机构3家，组建村镇银行、农商银行、担保小贷等机构21家。完成进出口总额24.9亿美元，年均增长9.4%。对外交流合作进一步加强，与国外6个城市缔结友好城市。

这五年，是我市社会建设不断加强、民生民利明显改善的五年。2015年全市城乡居民人均可支配收入达到16660元。五年新增城镇就业17.7万人，新增农村劳动力转移就业18.5万人。五大社会保险新增参保人数217万人，城乡低保、农村五保基本实现应保尽保，医疗救助年均覆盖26万多人次。累计减少贫困人口24.4万人。建设各类保障性住房19万套，完成农村危房改造4.6万户。安全生产形势持续稳定，综治维稳工作保持全省先进。教育均衡行动计划执行较好。人口和健康行动计划全面落实，基层医药卫生体制综合改革顺利完成。同步推进四级公共文化网络建设，基本形成了覆盖城乡的公共文化服务体系。其他各项社会事业全面发展。

在肯定成绩的同时，我们也清醒地看到，我市经济社会发展中还存在一些突出问题和“短板”，主要是：受全局性经济下行等因素影响，地区生产总值、规模工业增加值、社会消费品零售总额、进出口总额等指标增速没有达到预期；经济总量不大，产业结构不优；县域经济实力不强；财政收支矛盾突出；部分人民群众生活困难，扶贫攻坚任务艰巨，民生保障压力大；政府自身建设还存在一些薄弱环节，等等。这些都需要我们在今后的工作中认真加以解决，集中精力补短板促发展。

二、2015年工作回顾

2015年，我们坚持稳中求进工作总基调，强力推进大会战、大整治、大帮扶、大招商、大拆迁五大重点工作，攻坚克难，奋力拼搏，实现了经济的平稳增长和社会大局的和谐稳定。全市生产总值比上年增长8.5%；一般公共预算收入增长10.2%；固定资产投资增长18.5%；社会消费品零售总额增长12%；城乡居民人均可支配收入增长9.2%。

（一）三次产业稳步发展。农业生产平稳增长。实现农林牧渔业总产值388.7亿元，比上年增长4.5%。国家现代农业示范区建设有效推进。粮食产量稳步提高，生产面积稳定增加，蔬菜、黑茶、畜禽、水产品等产量产值均有不同程度增长。农业综合机械化水平达68.5%。农民专业合作组织发展到2509个。安化黑茶“全国知名品牌创建示范区”通过验收。工业经济稳中有升。扎实开展了“企业大帮扶”行动，规模工业增加值比上年增长7.5%。新增规模工业企业105家。高新技术产业实现增加值195亿元，增长12%。食品加工、电子信息等优势产业不断发展壮大。艾华集团成功在主板上市。第三产业较快发展。实现第三产业增加值533亿元，比上年增长11.7%。金融机构本外币存贷款余额分别为1344.3亿元和616.9亿元，存款余额较年初增加186.1亿元，各类贷款实际新增115.9亿元。电子商务加快发展，全市电商企业达7450家，高新区中南网商园获批国家电子商务示范基地。旅游产业稳步发展。成功举办了第三届中国湖南·安化黑茶文化节、湖南省第三届家具博览会和益阳市第六届房交会。

（二）项目建设扎实推进。切实抓好省市重点项目特别是洞庭湖生态经济区重大项目建设。大力推进项目征地拆迁工作，强化组织保障、司法保障和纪律保障。全市在建项目、新开工项目比上年分别增加439个和366个。交通建设大会战实现突破。完成交通建设投资120亿元，比上年增长33%，其中高速公路完成投资73.9亿元，增长95%。益阳南线高速建成通车。益娄、益马、南益、马安高速全面铺开建设；常安、安邵高速恢复施工；长益高速复线、安官、宁韶高速正在推进前期工作。干线公路、铁路、桥梁、站场和水运等项目进展顺利。（下转第2版）

园区建设大会战取得成效。大力实施“135”工程【1】，全市工业园区新建标准化厂房182.7万平方米，完成基础设施投资125.8亿元，新开工项目110个，新投产项目112个，引进创新创业企业300家，规模工业增加值增长8.8%，增速高出全部规模工业1.3个百分点。

（三）改革开放取得实效。各项改革全面推进。完成了9大类62项改革任务。市县政府机构改革基本完成，并乡并村工作有序开展。“四项清单”【2】制度、行政审批制度改革效果显著。现代农业改革试验全省率先试点。金融、农村、财税体制、商事制度【3】、医疗卫生体制、户籍制度、国有企业、城市管理和乡镇国土规划建设体制等改革顺利推进。引资争资成效明显。利用市域外资金形成固定资产投资376亿元，比上年增长12.2%。新引进项目285个，其中投资5亿元以上项目17个，引进了万达广场、海洋城等一批重大项目。争取上级各类财政性资金180亿元。对外贸易稳步增长。实现进出口总额5.9亿美元，新增30家外贸破零企业。

（四）城乡建设统筹推进。“一江三路”综合整治【4】扎实开展。启动了资江风貌带规划建设，以美化、绿化、亮化、序化为重点的“一江三路”综合整治工作取得重要成果。城乡规划不断健全。启动了《益阳市城市设计导则》和《益阳市美丽乡村导则》编制工作。完成了84个镇（乡）域村镇布局规划和130个农民集中居住区修建性详细规划。城镇建设加快推进。完成城镇基础设施建设投资98.8亿元，比上年增长21.9%，其中中心城区完成投资46.6亿元。全国文明城市、国家交通管理模范城市等创建工作有序开展。完成市政基础设施项目58个，城市生活垃圾焚烧发电项目主体工程已经建成，益阳大桥建设顺利推进。建成供水、供气和污水管网209.9公里，大通湖、灰山港、梅城污水处理厂建成运营。县城扩容提质和重点镇、特色小城镇建设扎实有效。城镇管理体制不断完善。围绕大益阳城市圈建设，进一步理顺了中心城区市区两级城市管理体制。整合了乡镇国土规划建设环保职能。新农村建设不断深化。美丽乡村建设扎实开展，13个美丽村庄、190个人居环境整治重点村建设进展顺利。农村清洁工程深入推进。建成农村公路286.8公里。完成239个村的农网改造。建成285处农村安全饮水工程，解决了94万人口的饮水安全问题。

（五）绿色发展力度加大。生态建设扎实推进。加强生态修复和生态保护，生态乡镇、生态村创建效果明显。完成植树造林23万亩，高效生态林业建设取得新成效。节能减排取得成果。万元规模工业增加值能耗下降9.5%，超额完成目标任务。加强重点减排项目建设，四项主要污染物总量减排全面完成目标任务。环境保护得到加强。扎实推进“两河”治理、大气污染防治、“矿山复绿”、农村环境综合整治等重点工作，加强环境监督执法，保证了环境安全。两型示范创建有序开展。两型综合示范区和两型标志性项目建设取得重大进展。

（六）民生事业全面进步。人民群众得到更多实惠。新增创业主体2.6万户，新增城镇就业3.6万人，新增农村劳动力转移就业2.4万人。五大社会保险新增参保6万人，启动了机关事业单位养老保险制度改革。城乡低保、农村五保、医疗救助等保障水平均有提高。精准扶贫扎实推进，减少贫困人口5.9万人。完成保障性住房建设4万套、农村危房改造6900户。15项重点民生实事全面完成。科技事业加快发展。完成专利申请2350件，实现专利授权1180件，3个项目获省科技进步奖。国家黑茶产品质量监督检验中心通过验收。教育事业均衡发展。建设义务教育合格学校92所，提质改造中小学校213所。卫生计生事业健康发展。积极实施基本公共卫生服务项目，群众看病就医更加便捷；大力推进城乡网格化服务管理，计生扶持政策有效落实。文体广新事业协调发展。文化惠民活动持续开展，文化遗产保护和利用取得成效。全民健身运动蓬勃发展。农村广播“村村响”第一期工程全面完成。其他事业全面发展。工商、质监、食品药品监管体制进一步理顺。对口援疆工作取得初步成效。统计、档案、审计、国防动员、人防、移民、民族宗教、对台、科协、地方志、地震、消防、气象、水文、烟草、住房公积金、妇女儿童、残疾人、红十字会、慈善等工作都取得了新的进步。

（七）社会大局和谐稳定。精神文明和民主法制建设得到加强。思想道德教育和精神文明创建不断深化，市民素质得到提高。全国法治先进城市创建活动有序推进，“六五”普法全面完成。基层民主政治建设不断加强，“四位一体”新型村级治理机制【5】逐步完善，和谐社区建设取得成效。安全生产形势持续稳定。扎实推进安全生产示范创建活动，狠抓安全生产责任落实和基层基础工作，强化隐患治理和常态化管理，没有发生重大及以上事故。综治维稳工作取得成效。持续深化平安益阳建设，社会治安防控体系不断完善。不断加强信访工作，有效解决了一批突出信访问题。认真落实维稳工作责任，扎实推行“五个特别”工作法【6】，社会大局保持稳定。

（八）自身建设不断加强。党风廉政建设取得新的成效。作风建设持续深化。扎实开展“三严三实”专题教育活动，认真执行作风建设有关规定，机关干部作风不断改进。认真履行党风廉政建设主体责任和监督责任，强化重点领域监督管理，严格执纪问责，严查腐败案件，政风行风进一步好转。依法行政水平稳步提高。依法全面履行政府职能，重大行政决策机制和行政执法体制逐步完善。自觉接受市人大及其常委会的法律监督、工作监督和市政协的民主监督，办理人大代表建议124件、政协委员提案106件，代表、委员满意和基本满意率达到98%以上。坚持以公开为原则、以不公开为例外，推动重大行政决策、财政预决算和“三公”经费等向社会全面公开。发展环境不断改善。加强政务服务，中心城区建设项目联合验收工作进展顺利，市政务中心审批服务事项实现应进全进。规范涉企检查收费行为，严厉打击强装强卸、无理阻工等违法行为，企业发展和项目建设环境逐步优化。

各位代表，成绩的取得是市委正确领导的结果，是市人大、市政协监督支持的结果，是全市上下团结奋斗的结果。在此，我代表市人民政府，向全市人民，向各民主党派、工商联、无党派人士、各人民团体和社会各界人士，向驻益人民解放军和武警官兵、政法干警，向中央、省属驻益单位，向热心支持益阳经济社会发展的各级领导和各界朋友，表示衷心的感谢！

“十三五”发展的总体要求和目标任务

根据党的十八届五中全会精神，市委五届七次全会通过了《中共益阳市委关于制定益阳市国民经济和社会发展第十三个五年规划的建议》。根据《建议》精神，市政府制定了《益阳市国民经济和社会发展第十三个五年规划纲要（草案）》。《纲要（草案）》已提请本次大会进行审议。

“十三五”的发展形势：“十三五”时期，我市进入全面小康社会建成期、经济扩量提质关键期、基础设施水平跃升期、发展动力活力迸发期和区域融合发展黄金期。综观国内外形势，全球经济仍处在国际金融危机后的深度调整期，我国经济发展进入中高速增长的新常态。在国家“四大板块”【7】和“三个支撑带”【8】战略新格局下，长江经济带、长江中游城市群、洞庭湖生态经济区等国家战略的实施，为我市更好地发挥优势、实现后发赶超拓展了空间，增添了动力。

“十三五”的指导思想：高举中国特色社会主义伟大旗帜，以邓小平理论、“三个代表”重要思想和科学发展观为指导，深入贯彻习近平总书记系列重要讲话精神，坚持“四个全面”【9】战略布局，贯彻创新、协调、绿色、开放、共享发展新理念，抢抓“一带一部”【10】、洞庭湖生态经济区和现代农业改革试验战略机遇，紧扣发展第一要务，把新型工业化作为后发赶超的第一选择，把项目建设作为加快发展的第一抓手，把民生改善作为富民强市的第一目标，突出实施大益阳城市圈和东接东进“两大战略”，继续推进交通和园区建设“两大会战”，促进“三量齐升”【11】，推动“五化同步”【12】，确保如期全面建成小康社会。

“十三五”的发展目标：地区生产总值年均增长9.5%，一般公共预算收入年均增长10%，固定资产投资年均增长18%，社会消费品零售总额年均增长10%，规模工业增加值年均增长11%，城乡居民人均可支配收入年均增长9%。到2020年全面消除绝对贫困。经济发展、资源环境、城乡建设、社会民生等其他预期性、约束性指标全面达到国省预期目标和全面小康要求。

“十三五”的发展战略：突出实施大益阳城市圈和东接东进战略。推进益沅桃一体化进程，加快构建以“1+3”城市组团为核心的大益阳城市圈，着力打造洞庭湖生态经济区南部中心城市。积极与长株潭城市群在规划、基础设施、产业、投资、消费、政务等方面实施对接，努力实现与长株潭城市群的融合发展、互补发展，着力打造长株潭都市区副中心城市。

“十三五”的发展格局：空间格局。规划形成“一体两翼三片区”的空间发展格局，即突出建设大益阳城市圈，同步推进湖区经济翼和山区经济翼的发展，逐步形成东接东进的大益阳城市圈核心片区、北联北通的北部发展片区和西连西拓的西部发展片区。产业布局。形成“一横两纵”的产业发展轴线。以长益常经济走廊为横向主轴线，主要布局装备制造、电子信息、生物医药、新材料和电子商务、文化创意、健康养老等现代服务业。纵向形成以益沅南组成的湖区经济翼，主要布局装备制造、食品加工、船舶、水产养殖、休闲观光农业和湿地渔耕文化旅游等；以益桃安组成的山区经济翼，主要布局新能源、新材料、竹、茶、中药材和生态农耕文化旅游等。城镇布局。围绕“两中心两城市”【13】的定位，推进益沅桃一体化，加快推进东部新区两型示范区建设。做大做强县（市）城，积极发展中心镇、重点镇、特色小城镇，构建结构合理、布局协调、功能互补的城镇体系。交通布局。加快构建综合交通体系，着力打造区域性综合交通枢纽城市，建立以“一环七射”【14】、“三纵一横”【15】高速公路为主骨架的公路网络，逐步形成益阳－长沙－上海、益阳－荆州－北京、益阳－常德－成都、益阳－娄底－广州等四大高速公路通道。建立“三纵四横”【16】的铁路基础设施网络，争取尽快启动渝长厦铁路常益长段高铁建设。建立“一纵两横”【17】的千吨级航道水运网络。推进通用机场建设和城市轨道交通线网规划。

“十三五”的发展任务：按照“五化同步”的要求，突出抓好“五大建设”。一是强力推进产业建设。坚持“产业兴市、工业强市”，按照“扩总量、提质量”的要求，大力推进“提一扩二加三”【18】，着力构建以新型工业为主导、现代农业为基础、现代服务业为重要支撑的现代产业新体系。二是强力推进基础设施建设。积极适应民生改善和经济发展要求，全面推进交通、水利、能源、信息等基础设施“四张网”建设，进一步夯实发展基础。紧紧围绕大益阳城市圈建设这个核心，大力加强城镇基础设施建设，增强城镇承载能力，提升新型城镇化水平。三是强力推进民生建设。坚持以增进人民福祉为目标，以推进精准扶贫为重点，不断加大民生投入，着力办好民生实事，扎实抓好就业创业、社会保障、安全生产、综治维稳等各项工作，全面发展教育、卫生、文化、体育等民生事业，努力实现基本公共服务均等化，使全市人民过上更加幸福美好的生活。四是强力推进生态文明建设。不断完善主体功能区制度，全面推进两型社会建设，加快推动生产方式和生活方式的绿色化；依托洞庭湖生态经济区建设这个大平台，进一步加强自然山体水体保护，切实抓好水生态建设与水污染防治，加快推进国家生态文明示范市创建，坚定不移走生产发展、生活富裕、生态良好的文明发展之路。五是强力推进体制机制建设。冲破思想观念束缚，突破利益固化藩篱，全面深化各项改革，建立健全有利于加快发展的体制机制。坚持以经济体制改革为重点，大力推进市场运行、行政管理、规划建设、投资融资、招商引资、就业创业、文化科技、生态文明、精准扶贫等体制机制改革创新，做强发展动力，激活发展活力。

2016年工作安排

2016年，全市上下要认真贯彻落实中央、省委和市委经济工作会议精神，以“十三五”规划为引领，主动适应经济发展新常态，坚持稳中求进总基调，坚持改革开放，坚持供需两侧【19】着力，坚持稳增长、调结构、惠民生、防风险，继续强力实施大会战、大整治、大帮扶、大招商、大拆迁，推进全市经济社会持续健康平稳发展，为建成全面小康社会，建设富饶美丽幸福新益阳开好局、起好步。

2016年经济社会发展的主要目标是：生产总值增长9.5%，一般公共预算收入增长10%，固定资产投资增长20%，社会消费品零售总额增长12%，规模工业增加值增长10%，城乡居民人均可支配收入增长9.5%，单位生产总值能耗、主要污染物排放量、人口自然增长率控制在省定目标以内。

为完成上述目标任务，要突出抓好以下十项工作：

一、以加速产业扩张和升级为着力点，强力推进新型工业化

坚持把新型工业化作为经济增长的关键，全力打造新型工业城市。扩张工业经济总量。强力实施大产业、大企业、大集群、大园区、大品牌战略，着力构建“大企业顶天立地、小企业铺天盖地”的企业发展格局，推动工业经济总量快速提升。按照打造千亿产业的要求，支持食品加工、装备制造、新能源、电子信息等优势产业做大做强，同时培育发展一批百亿产业、百亿集群、百亿园区、百亿企业。积极支持以安化黑茶产业、沅江船舶产业、桃江竹木产业、资阳槟榔产业等为重点的特色县域经济发展。按照“储备一批、建设一批、投产一批”的要求，扎实推进重大产业项目建设。推动工业经济转型升级。加快发展新材料、生物医药、节能环保等战略性新兴产业，确保战略性新兴产业增加值占生产总值的比重比上年提升1个百分点。加强对纺织、冶金、建材、造纸、竹木加工等传统产业的技术改造，力争工业技改投资比上年增长12%以上。实施创新驱动发展战略，强化企业技术创新主体地位，确保高新技术产业增加值增速高于全省平均水平。继续强力推进园区建设大会战。认真落实“135”工程目标任务，新建标准化厂房150万平方米，已建标准化厂房利用率达80%以上，引进创新创业企业200家以上，完成基础设施投资100亿元。强化园区招商和项目落地工作，实现园区新开工、新投产项目各100个。加强高新区东部产业园汽车零配件生产基地、长春电子产业园、资阳新材料产业园、沅江船舶产业园及服装产业园、赫山中医药产业园等专业园区建设，引导产业集聚式发展。广泛深入开展企业帮扶行动。落实中央去库存、降成本等供给侧结构性改革政策措施，围绕帮减负、帮营销、帮环境、帮项目、帮融资五个重点，在全市范围内深入开展企业帮扶行动，对重点企业逐一分析、逐一研究、逐一扶持，帮助企业解决各类实际问题，破解发展瓶颈。发展私募股权基金，积极推动企业通过主板、创业板和新三板上市融资.

二、以大益阳城市圈建设为核心，加快推进新型城镇化

认真贯彻落实中央城市工作会议精神，坚持以人民为中心的发展思想。紧紧围绕大益阳城市圈建设，不断提升城市环境质量、人民生活质量和城市竞争力。突出抓好大益阳城市圈规划。重点编制完成大益阳城市圈发展战略规划和中心城区道路、地下空间建设利用、地下综合管廊建设、海绵城市【20】、园林城市等专项规划。切实强化规划执行，坚决维护规划的权威性和严肃性。着力加快大益阳城市圈建设。加强城市道路桥梁建设。按照“多环多快多射”【21】的原则，启动青龙洲大桥前期工作，开工建设白马山路延伸线、虎山路、关山路，打通大益阳城市圈的重要交通环线。继续推进益阳大桥、益阳大道西延线、东部新区内外环线、银城大道、高新大道建设，抓好梓山东路、海棠西路、教育路、团山路等城区道路通畅工程建设。全面启动资江风貌带建设。以“一水”、“两岸”、“两路”、“五桥”【22】、“十园十景”【23】建设为重点，以打造产业带、生态带、休闲带、文化带为目标，致力建设国内外知名的城市滨江风貌带。率先启动防洪堤和滨江路建设。抓好公园绿地建设。按照5A级景区标准启动梓山湖生态公园建设，加快资阳公园建设，进一步提质改造会龙山公园，建设一批街头绿地。加强“两供两治”【24】设施建设。启动第二水源地前期工作，建设益阳市第四水厂；抓好气化益阳天然气管道建设；加快城乡生活垃圾一体化工程建设，完成垃圾焚烧发电项目建设和污水处理厂提标改造；抓好城市地下综合管廊建设；推进中心城区雨污分流及黑臭水体整治。加快“两房两棚”【25】项目建设。完成保障房建设4.6万套（户），其中中心城区完成棚户区改造1.5万户，突出抓好资江两岸、会龙山片区、明清古城片区、梓山湖片区、毛家塘村改造示范工程。切实强化大益阳城市圈管理。继续推进全国文明城市、国家交通管理模范城市、国家园林城市、国家环保模范城市、国家历史文化名城等系列城市创建（申报）活动。加快“智慧城管”、“法治城管”建设，实施城市管理“六大专项整治行动”【26】，提高城市管理的法治化、智能化和精细化水平。创新大益阳城市圈体制机制。加强大益阳城市圈建设的组织领导和统筹协调。出台加快大益阳城市圈建设的决定。全面推进大益阳城市圈规划建设管理体制机制创新。以“一江三路”综合整治和建设为突破，重点抓好益沅桃基础设施、产业布局、公共服务、环境保护等领域一体化工作。启动实施大益阳城市圈公交一体化。启动益沅桃城市群区域规划条例立法工作。不断加强县城和小城镇规划建设管理。加快城乡各类规划编制，尽快实现城乡规划全覆盖。实施县城扩容提质工程，加快中心镇、重点镇和特色小城镇建设，构建多点支撑的城镇体系。探索推进马武融合发展。继续抓好国家、省、市级卫生乡镇创建活动。引导县城和小城镇进一步完善管理体制，提升管理水平。

三、以“互联网+”为引领，大力推进信息化

制定益阳“互联网+”行动计划，发挥“互联网+”的乘数效应，加快新一代信息技术成长，推进信息化迈上新台阶。加快发展“互联网+产业经济”。推进“互联网+制造”、“互联网+农业”、“互联网+物流”等产业发展模式，引导安化黑茶、大通湖大闸蟹、沅江芦笋、桃江竹制品等特色产业通过互联网拓展市场、加快发展。抓好鱼形山智慧谷和智慧旅游项目建设前期工作。积极发展电子商务和服务外包产业，抓好桃江县全国第二批电子商务进农村示范县和中南网商园等项目建设。全面推进“互联网+民生事业”。加强信息惠民工程建设，推进基于互联网和移动终端的社区管理、医疗卫生、就业创业、社会保障、教育培训等民生事业发展。抓好全民阅读数字平台、食品药品监管信息惠民工程、智慧校园建设。鼓励各种市场主体共同参与“互联网+”民生项目，让群众共享“互联网+”发展成果。扎实做好“互联网+政务服务”。整合互联网政务信息资源，推进政务服务信息化建设。重点建设云计算【27】中心、信息资源共享平台、公共安全视频监控系统、一卡通等项目，打造高效便捷、互联互通的政务服务体系。加快建设突发公共事件预警信息发布系统，尽快启动中心城区数字化城市管理系统平台建设。积极拓展“互联网+招商引资”。大力引进IT企业、大数据中心、网络代理商，精心打造互联网招商平台。不断夯实“互联网+”发展基础。推进城市基础网络、宽带乡村、物联网【28】应用示范等重大工程。推动光纤入户和宽带网络改造升级，提高城乡宽带网络普及水平和接入能力。大力推进4G网络和无线城市建设。实现全市85%以上的行政村光网覆盖，100%的行政村无线互联网覆盖。

四、以现代农业改革试验为突破，统筹推进农业现代化和新农村建设

加快国家现代农业示范区建设。围绕创新现代农业产业体系、构建新型农业经营体系等9大改革重点，稳步推进现代农业改革试验，全面提高农业综合生产能力、抗风险能力和市场竞争能力，促进农业增效和农民增收。提升农业产业化水平。继续推进土地有序流转，加快培育新型农业经营主体，发展适度规模经营，推进农业生产经营专业化、规模化、集约化。不断提升农业机械化水平，继续完善农业社会化服务体系。大力发展农产品精深加工，支持农业产业化龙头企业做大做强。启动智慧农业园和肉类深加工基地等项目建设。提高粮食安全保障能力。进一步强化粮食主产区地位，坚持最严格的耕地保护制度，严守基本农田红线。全面实施粮食增产提质计划，加大高标准农田建设力度，落实粮食财政奖补政策，稳定粮食生产面积和粮食总产量。做大做强农业特色产业。切实加强蔬菜、茶叶、花卉苗木、笋竹、芦笋、水产、中药材等农业特色产业发展。抓好茶叶标准园、笋竹两用林和标准化规模养殖等基地建设。探索发展稻虾共生种养模式。保障农产品质量安全。大力开展农业标准化、品牌化建设及农产品质量安全专项整治等行动，加强农产品质量安全检验检测和动植物疫病防控能力建设，确保不发生重大农产品质量安全事故和重大动植物疫情。加快建设美丽乡村。加强对农民建房的引导、管理和服务，启动实施规范农民建房地方立法工作。按照“益阳传统、时代精神”的要求，坚持“经济、美观、实用、特色”的原则，积极推广以“青瓦顶、花格窗、封火墙、穿斗架、白灰色”为标志的现代美丽民居建设。深入推进农村清洁工程，扎实开展农村垃圾5年综合治理，完成190个行政村人居环境整治和19个美丽乡村示范村建设。加快大湖生活垃圾焚烧发电项目建设。

五、以交通建设为先导，全面推进基础设施建设

坚持不懈推进交通、水利、能源等基础设施建设。继续强力推进交通建设大会战。力争完成交通基础设施投资130亿元，其中高速公路投资100亿元。抓好高速公路建设，确保常安、安邵高速益阳段建成通车；加快益娄高速益阳段、南益、益马、马安高速建设进度，新开工建设长益高速复线、安官高速益阳段；完成益平、宁韶、张新高速益阳段前期工作。加强干线公路建设，新开工建设S221南益高速大通湖互通至河坝段、S228资阳段、S217金盆至南大、G353浪拔湖镇改线工程、S324东坪至梅城和牛田至大福、S322乐园至漉湖等干线公路；确保S225安化段、S224梅城至黄柏界、S217河坝至金盆建成通车；加快推进S228沅江段、S230杨林坳至高桥等项目建设；启动G319益阳段改造的前期工作。推进桥梁、站场和水运工程建设，加快安化大桥建设，认真实施渡改桥、危桥改造、客运站、招呼站等项目，加快推进千吨级港区码头、南茅运河航道整治工程等项目建设。完成农村公路通畅工程750公里。积极做好渝长厦铁路常益长段高铁、常岳九铁路、洛湛铁路益娄段扩能改造等项目前期工作，力争今年基本确定建设方案。启动益阳通用机场前期工作。强力推进水利基础设施建设。抓好沟渠清淤疏浚项目；启动长春垸、大通湖垸等河湖连通工程。加强城市防洪工程和共双茶垸等堤垸安全工程建设，加快实施大中型病险水闸除险加固工程，以及资江干流、中小河流重要河段治理、河道保洁等项目。巩固提升农村饮水安全工程，加强水质保障能力建设。实施桃花江、廖家坪水库灌区节水配套改造工程，加快小型农田水利建设。启动新华水库水利枢纽前期工作。不断完善气象灾害防御体系。强力推进能源基础设施建设。重点支持桃花江核电项目建设，进一步加强核电项目科普和公众沟通工作。加快沅江漉湖、安化天子界、芙蓉山、桃江邱家仑、松木塘风电场建设进度，启动大通湖大湖水上光伏发电、舵杆洲风电场项目。大力实施全球能源互联网发展战略和特高压电网建设工程，继续抓好农网升级改造和城网建设。做好安化抽水蓄能发电等项目前期工作。

六、以培育新兴业态为主攻方向，着力推进现代服务业发展

把握现代服务业发展的良好机遇，推动现代服务业向高端化、智能化、绿色化方向发展。大力发展新兴服务业。积极培育电子商务、文化创意、科技服务、节能环保、融资租赁等各类新兴服务业态，着力引进一批带动作用明显的服务业企业。积极推进湖南（益阳）工艺美术创意设计园、万国荟进出口商品城、芙蓉云计算数据中心、品尚电商园等重点服务业项目。大力发展休闲旅游业。围绕“文化、生态”两大主题，打响休闲益阳品牌，着力构建现代休闲旅游产品体系，积极创建国家旅游休闲示范城市。围绕湖、茶、竹等益阳特色，精心打造周末旅游、假日旅游、短途旅游精品线路。突出抓好以中心城区历史文化、佛教文化、三国文化为重点的文化旅游，以梓山湖片区、清溪村、皇家湖、南洞庭湖等为重点的生态旅游。着力引进旅游开发战略投资者，加快旅游景区景点开发和提质，努力创建一批5A级景区。继续提升茶马古道、桃花江竹海、梅山文化生态园等景区品质，加快推进游客服务中心、江南古城等重点旅游项目建设。切实加强旅游基础设施与配套设施建设，不断改善“吃住行游购娱”等要素条件。积极培育以大健康产业为主的生活性服务业。加快“健康益阳”建设，培育大健康产业，促进养老服务、养生休闲、健康服务等产业快速发展。支持康雅医院养老项目建设。全面提升餐饮、住宿、家政、物业等生活性服务业的发展水平。落实中央去库存的政策措施，促进房地产市场稳定发展。大力发展生产性服务业。积极支持现代金融业发展。加强银行业机构建设，推进金融产品与服务创新，积极开展“金融服务创新示范园”创建活动。优化金融生态，防控金融风险。支持桃江县做好全国农村土地经营权抵押贷款试点工作。力争全年新增各类贷款100亿元。加快现代保险业发展。大力发展现代物流业，积极支持海吉星、益阳（湘运）现代物流园、兰溪粮食物流园、南洲物流园、安化黑茶物流中心、红联冷链等项目建设。大力发展现代商贸流通业，积极支持万达广场、海洋城、顺德城等项目加快建设。

七、以精准扶贫为重点，协调推进社会民生建设

坚持把改善民生作为政府工作的出发点和落脚点，努力实现共享发展。强力推进精准扶贫。坚持以精细化、差异化、社会化扶贫为重点，加强贫困地区的基础设施、基本公共服务、基础产业和基层组织建设，加大资金、政策、项目等扶持力度，健全金融服务、社会参与、责任考核、建档立卡、贫困对象退出等工作机制，构建专项扶贫、行业扶贫、社会扶贫“三位一体”工作格局，实现精准识贫、精准扶贫、精准脱贫。不断加强贫困村“六大建设”【29】，强化扶贫工作“六项举措”【30】，认真落实“六个一批”【31】，实现8.4万贫困人口脱贫。认真抓好创业就业和社会保障。大力推进大众创业、万众创新。统筹抓好高校毕业生、农村转移劳动力、城镇困难人员、退役军人就业。实现新增城镇就业3.4万人，新增农村劳动力转移就业1.8万人，新增创业主体2.3万户，城镇登记失业率控制在4.5%以下。启动全民参保登记计划，努力扩大社会保险覆盖面。继续推进机关事业单位和城乡居民养老保险制度改革。做好降低社保费率的衔接工作。健全被征地农民社会保障和安置办法、城乡居民大病保险体系及补偿办法。继续抓好城乡低保、农村五保、临时救助等社会救助体系建设。协调推进各项社会事业。促进文化体育事业繁荣与发展。启动实施和高标准建设益阳文化中心。结合资江风貌带建设，精心打造“一江两岸”益阳历史文化走廊。启动兔子山遗址公园项目前期工作，完成明清古巷保护性修缮，整理出版《黄自元书法全集》。倡导全民阅读行动，推动书香益阳活动【32】深入开展。继续抓好花鼓戏振兴工程，办好“益阳花鼓大戏台”。加快基层综合性文化服务中心建设。支持发展新闻事业。扎实推进广播“村村响”二期工程和电视“户户通”工作。倡导全民健身运动，提升竞技体育水平。推动教育均衡发展。深化教育综合改革，加强教师队伍建设，着力提高教育质量。加强校园安全隐患排查治理。启动标准化学校建设，落实城区学校规划布点，完成迎宾路小学和龙洲小学建设，启动马良学校、碧桂园小学、丁香学校建设。实施农村中等职业教育“三年攻坚计划”【33】和特殊教育提升计划。鼓励社会力量办学。大力发展高等教育。做好省第三轮“两项督导评估考核”【34】工作。强化公共卫生建设。落实传染病、地方病及职业病防治规划和行动计划，抓好重大疾病防控。强化妇幼健康、中医中药、社区医疗保障，提高全民健康保障水平。深入开展爱国卫生运动。巩固和完善基本药物制度改革。强化食品药品安全管理和风险监测，推进食品药品安全信息化。加快市中心医院急诊医技大楼、市妇幼保健院等项目建设。依法组织实施二孩政策和社会抚养费征收，继续推进城乡网格化服务管理，提升计生优质服务水平。加快科技事业发展。大力推进科技创新，加强知识产权保护，促进科技成果转化，申报建设省级农业科技园。积极发展其他各项事业。继续大力实施人才强市工程，完善人才引进、培养、使用的科学机制，为创新发展提供人才和智力保障。强化质量建设和质量安全监管。进一步加大市场监管力度，维护公平公正的市场秩序。深入开展国防动员工作，深化全民国防教育，推动军民融合深度发展。支持工会、共青团、妇联等群团组织依照章程开展工作。切实抓好国家安全、民族宗教、外事侨务、移民、对台、人防、统计、审计、供销、档案、保密、地方志、老龄、残疾人、住房公积金、红十字会等各项工作。加强和创新社会治理。深入推进文明创建活动，不断提高公民道德素养。加快全国法治先进城市创建进程；高标准启动“七五”普法工作，推进公共法律服务中心和社区矫正中心建设。稳步推进社区民主建设，继续开展和谐社区创建。扎实抓好合并705个村的工作。进一步完善“四位一体”新型村级治理机制，加快培育村级集体经济。继续抓好“天网工程”【35】，提升治安防控科技化水平。健全以民意为导向的社会治安重点整治机制。不断深化“三调联动【36】化矛盾、息访息诉促平安”专项活动，落实信访维稳工作责任，营造和谐稳定的社会环境。着力抓好安全生产工作。继续围绕“夯实基础年”主题，强化安全监管，落实安全责任，突出隐患整治和打非治违两个重点，推进专项整治，保持全市安全生产形势持续稳定向好。

八、以洞庭湖生态经济区建设为抓手，深入推进生态文明建设

全面落实《洞庭湖生态经济区规划》，坚持走绿色、低碳、循环发展之路，实现全面协调可持续发展。大力发展生态经济。推动绿色清洁生产、资源综合利用，广泛开展传统制造业绿色化改造，加快推广节能环保新技术、新装备、新产品，着力构建绿色产业体系。推动省级循环经济试点示范市（县）、示范园区、示范企业的申报工作。持续加强生态修复与保护。深化国家生态文明示范市创建。加强对湿地、耕地、森林的生态修复。加快水资源保障、水生态修复、水安全运行等重大工程建设，推进“百湖湿地”修复、南茅运河生态走廊等生态项目。加强森林资源保护管理。启动城市规划区自然山体水体保护和利用管理条例立法工作。切实加强污染防治。抓好柘溪水库污染整治和大通湖水环境治理。推进农村面源污染治理，加强工业源、生活源和大气污染防治。继续抓好“两河”治理和中心城区“两年治污计划”的落实。切实抓好重金属污染防治、农村环境综合整治等项目建设。强化减排项目落实，全面完成年度减排目标任务。抓好“两型”示范创建。建立全市两型社会建设综合评价制度。着力抓好东部新区建设，加快完善基础设施，理顺管理体制机制。全面推进两型示范单位创建和十大两型标志性项目【37】建设。

九、以增强发展动力和活力为目标，稳步推进改革开放

坚持改革与开放相结合，以开放促改革、以改革促发展，加快构建要素有序流动、资源高效配置、市场深度融合的开放型经济新体制。全面深化各项改革。突出抓好供给侧结构性改革【38】。纵深推进已经启动的各项改革，深化行政管理体制、科技体制、文化体制、医疗卫生体制、财税体制、国企国资、金融、农村、农垦、户籍制度、不动产登记制度、供销合作社等改革工作，充分释放改革红利。全力推进招商引资。突出“招大招群招特”的工作思路，强化“精准招商”理念，坚持“管行业必须管招商”的原则，创新招商引资工作机制，配齐配强招商引资力量，落实招商引资目标任务，突出抓好行业招商、平台招商、专业招商，加强招商引资分类管理、分类考核，确保招商引资取得重大突破。研究制定企业抱团进入、集群引进的优惠政策，提升产业集群承接能力。着力引进战略投资者，重点引进一批世界（国内）500强、央企、行业龙头企业和上市公司。加强对外贸易和对外交流。积极落实国家扩大出口鼓励政策，建立出口担保体系，培育出口知名品牌，增强出口产品竞争力。重点支持艾华电子、太阳鸟游艇、益华水产等企业参与国际竞争。加快口岸机构建设，确保商检大楼正式投入运营，争取设立海关益阳办事机构。继续加强与国外友好城市的交流与合作。

十、以持续改进工作作风为保障，扎实推进政府自身建设

认真贯彻落实中共中央、国务院《法治政府建设实施纲要（2015－2020）》，着力建设法治政府、创新政府、廉洁政府和服务型政府，努力提升政府公信力和执行力。深入转变工作作风。巩固群众路线教育实践活动和“三严三实”专题教育成果。强化责任担当，提升能力水平，有效传导压力，努力解决不想为、不敢为、不善为、不作为的问题。强化行政效能建设，健全更科学高效的绩效评估考核体系，确保政府各项工作高效有序运转，用政府的勤政指数换取百姓的幸福指数。切实转变政府职能。坚持政企分开、政资分开、政事分开、政社分开，进一步理顺政府与市场、政府与社会的关系，切实强化政府的市场监管、社会管理、公共服务、环境保护等职责。持续深化行政审批制度改革，严格推行“四张清单”制度并实行动态管理，不断激发市场活力。大力转变行政方式。全面推进依法行政。完善重大行政决策程序制度，增强公众参与实效，提高专家论证和风险评估质量，严格决策责任追究，推进行政决策科学化、民主化、法治化。深化行政执法体制改革，推行行政执法公示制，落实行政执法责任制，加大关系群众切身利益的食品安全、环境保护、安全生产等重点领域执法力度，提升行政执法的社会满意度。加强政务公开，优化政务服务。启动市政务服务中心建设，完善公共资源交易平台，抓好市长热线平台整合，推进村级综合服务平台建设。切实强化对行政权力的制约和监督，严格执行人大及其常委会的决议决定，积极推进协商民主，认真办理人大代表建议和政协委员提案，主动接受舆论监督和群众监督。加快转变思想观念。牢牢把握解放思想、科学发展这一理念，切实增强忧患意识，进一步转变观念、拓宽视野，破除小进即满、小富即安的保守思想，树立敢闯敢干、敢为人先的进取精神，营造抢抓机遇、破旧立新、迎难而上的良好氛围，凝聚广大干部群众的积极性和创造性，形成推动益阳跨越发展的强大合力。持续推进党风廉政建设。深入开展党风廉政建设和反腐败工作，认真学习贯彻“一准则、两条例”，严格落实党风廉政建设主体责任和监督责任，强化制度约束，大力正风肃纪，努力营造风清气正的良好环境。

各位代表，新的时代赋予了我们新的使命，新的征程需要我们奋勇开拓。让我们在中共益阳市委的坚强领导下，抢抓发展机遇，合力攻坚克难，锐意进取，奋发有为，为加快建成全面小康社会而努力奋斗！
